# A novel anoikis-related prognostic signature associated with prognosis and immune infiltration landscape in clear cell renal cell carcinoma

**DOI:** 10.3389/fgene.2022.1039465

**Published:** 2022-10-19

**Authors:** Zhuo Chen, Xiao Liu, Zhengjie Zhu, Jinchao Chen, Chen Wang, Xi Chen, Shaoxing Zhu, Aiqin Zhang

**Affiliations:** ^1^ The Cancer Hospital of the University of Chinese Academy of Sciences (Zhejiang Cancer Hospital), Institute of Basic Medicine and Cancer (IBMC), Chinese Academy of Sciences, Hangzhou, China; ^2^ Shaoxing TCM Hospital Affiliated to Zhejiang Chinese Medical University, Shaoxing, Zhejiang, China

**Keywords:** clear cell renal cell carcinoma, anoikis-related genes, risk model, immune infiltration landscape, prognosis, consensus clustering

## Abstract

**Background:** Clear cell renal cell carcinoma (ccRCC) is the most common histological subtype of renal cell carcinoma (RCC). Anoikis plays an essential function in tumourigenesis, whereas the role of anoikis in ccRCC remains unclear.

**Methods:** Anoikis-related genes (ARGs) were collected from the MSigDB database. According to univariate Cox regression analysis, the least absolute shrinkage and selection operator (LASSO) algorithm was utilized to select the ARGs associated with the overall rate (OS). Multivariate Cox regression analysis was conducted to identify 5 prognostic ARGs, and a risk model was established. The Kaplan-Meier survival analysis was used to evaluate the OS rate of ccRCC patients. Gene ontology (GO), Kyoto encyclopedia of genes and genomes (KEGG), and Gene set enrichment analysis (GSVA) were utilized to investigate the molecular mechanism of patients in the low- and high-risk group. ESTIMATE, CIBERSOT, and single sample gene set enrichment analysis (ssGSEA) algorithms were conducted to estimate the immune infiltration landscape. Consensus clustering analysis was performed to divide the patients into different subgroups.

**Results:** A fresh risk model was constructed based on the 5 prognostic ARGs (*CHEK2, PDK4, ZNF304, SNAI2, SRC*). The Kaplan-Meier survival analysis indicated that the OS rate of patients with a low-risk score was significantly higher than those with a high-risk score. Consensus clustering analysis successfully clustered the patients into two subgroups, with a remarkable difference in immune infiltration landscape and prognosis. The ESTIMATE, CIBERSORT, and ssGSEA results illustrated a significant gap in immune infiltration landscape of patients in the low- and high-risk group. Enrichment analysis and GSVA revealed that immune-related signaling pathways might mediate the role of ARGs in ccRCC. The nomogram results illustrated that the ARGs prognostic signature was an independent prognostic predictor that distinguished it from other clinical characteristics. TIDE score showed a promising immunotherapy response of ccRCC patients in different risk subgroups and cluster subgroups.

**Conclusion:** Our study revealed that ARGs play a carcinogenic role in ccRCC. Additionally, we firstly integrated multiple ARGs to establish a risk-predictive model. This study highlights that ARGs could be implemented as a stratification factor for individualized and precise treatment in ccRCC patients.

## Introduction

Renal cell carcinoma (RCC) is the most common malignancy in the urinary system, affecting over 430,000 newly diagnosed cases and 170,000 deaths in 2020 worldwide (Sung et al., 2021). Clear cell renal cell carcinoma (ccRCC) is the most common histological subtype, occurring in approximately 75% of RCC (Choueiri and Motzer, 2017). Although new strategies have greatly improved life expectancy and quality of life in patients with advanced ccRCC, the prognosis of metastatic RCC patients is still unsatisfactory, with the 5-year survival rate remaining less than 15% (Klatte et al., 2018). Therefore, investigating novel diagnostic biomarkers and prognostic model is vital for the clinical management of ccRCC.

Disruption of cell-cell attachment or cell-ECM attachment leads to a form of apoptosis called “anoikis” (Raeisi et al., 2022). This process can eliminate misaligned or shed cells under physiological or pathological conditions, contributing to the realization of tissue homeostasis. Anoikis is involved in several pathological processes, including carcinogenesis. After a continuous separation process from each other or the ECM, cancer cells metastasize, migrate to remote endpoints, reattach, and proliferate in new sites, resulting in tumor spread and loss of surgical opportunities (Guan, 2015). Cancer cells employ several mechanisms to eliminate anoikis, promoting their invasiveness and metastasis. By promoting oncogenic signals that induce pro-survival pathways, or changes in the acidic environment in the tumor microenvironment and reactive oxygen species (ROS) generation, cancer cells have a great impact on promoting anti-anoikis (Wang C. et al., 2019; Hu et al., 2019; Vander Linden and Corbet, 2019). Anoikis also has potential therapeutic value in RCC. In ccRCC, interference with TIM-3 protein expression can attenuate the invasion ability by aggravating anoikis (Yu et al., 2017). Knockdown of anoikis-related protein Tryptophan 2,3-dioxygenase (TDO2) inhibits the proliferation and invasion of RCC cells and may be a promising marker for RCC targeted therapy (Pham et al., 2021). Quinazolines trigger anoikis in RCC by targeting the focal adhesion survival signaling, resulting in potent antitumor effects (Sakamoto et al., 2011). Recent studies have shown that the ECM deprivation system (EDS) based on Fibronectin (FN) -targeted self-assembling peptide can effectively inhibit renal cell carcinoma by reversing anoikis resistance (Wang et al., 2022). However, there is no effective RCC risk prediction model based on anoikis to reflect the impact of anoikis-related genes on prognosis comprehensively.

In this study, following the exploration of The Cancer Genome Atlas database (TCGA) database, the correlation between ARGs and clinicopathological characteristics of ccRCC patients was systematically investigated. A novel risk model was established based on 5 prognostic ARGs, and the capability of ARGs in predicting the prognosis of patients with ccRCC was further evaluated. Moreover, the immune infiltration of ccRCC patients and the possible signaling pathways involved were comprehensively explored in this study. This study aimed to provide novel insights and perspectives into a new potential therapeutic strategy and antitumor targets for ccRCC.

## Materials and methods

### Data collection

The transcriptome matrix and clinical materials were downloaded from The Cancer Genome Atlas database (TCGA) (https://portal.gdc.cancer.gov/). The samples without survival time or the survival time less than 0 were excluded, and a total of 525 ccRCC samples were included for the subsequent analysis in this study. Perl scripts were utilized to extract the transcriptome matrix of each ccRCC sample and merged for further analysis. The symbol of mRNAs was annotated using the ensembles human genome browser GRCh38. p13 (http://asia.ensembl.org/index.html). Clinical materials, including age, gender, grade, stage, and TMN stage were collected from the TCGA database *via* Perl scripts. All information and clinical matrix involved were downloaded from the public database. Approval from the ethics committee and written informed consent from patients were not required.

### Identification of anoikis-related genes and risk model construction

The anoikis-related genes (ARGs) were collected from the Molecular Signatures Database (MSigDB database) (https://www.gsea-msigdb.org/gsea/). A total of 34 ARGs were identified to evaluate the prognosis value for ccRCC (Supplementary Table S1). According to the univariate Cox regression analysis, the least absolute shrinkage and selection operator (LASSO) algorithm was utilized to select the ARGs associated with the overall survival (OS) rate *via* the R package “glmnet”. Next, a multivariate Cox regression analysis was conducted to select the ARGs which could independently predict the prognosis for ccRCC, and a risk model was established based on the prognostic ARGs. The risk model was constructed according to the ARGs prognostic signature using the formula: (0.719 x the expression of *CHEK2*) + (−0.171 x the expression of *PDK4*) + (−0.725 x the expression of *ZNF304*) + (0.413 x the expression of *SNAI2*) + (0.479 x the expression of *SRC*). Based on the median risk score, the samples with ccRCC were divided into low-risk and high-risk groups. The Kaplan-Meier survival curve was employed to evaluate the OS rate of ccRCC patients in the low- and high-risk group using a log-rank algorithm *via* R package “survival”. Principal component analysis (PCA) score plot was used to investigate the distribution pattern of the patients in the low- and high-risk group *via* the R package “ggplot2”.

### Internal validation of risk model

Based on the ARGs, 525 ccRCC samples in the TCGA database were classified into the training cohort and test cohort with a ratio of 7:3 based on R package “caret” (Tsiliki et al., 2015). A total of 368 samples were divided into training cohort and 157 samples were divided into test cohort. The risk score of each sample was calculated according to the formula, and the samples were divided into low- and high-risk group according to the median risk score in the both cohorts.

### Independent prognosis analysis of risk model

Univariate and multivariate Cox regression analysis were utilized to evaluate the independence of the risk model *via* the R package “survival”. The nomogram model was established of the clinicopathological characteristics and risk score *via* the R package “rms”. Based on the Cox regression analysis, all variates were calculated and evaluated the 1-. 3-, and 5-year survival probability of ccRCC. Calibration diagram was a common parameter to assess the accuracy of nomograms. R package “pROC” was used to evaluate the diagnostic accuracy of the risk score and clinicopathological characteristics for ccRCC. The prognostic capability of the risk model at 1-, 3-, and 5- years was validated using time-dependent receiver operating characteristic (ROC) analysis *via* R package “timeROC”.

### Consensus clustering analysis

Based on the 5 prognostic ARGs, the consensus clustering was performed with max K = 9 *via* R package “ConsensusClusterPlus.” The clustering was established on the grounds of partitioning around medoids with “euclidean” distances, and 1000 verifications were performed. Next, according to the optimal classification of K = 2–9, the patients with ccRCC were cluster into different molecular subtypes for further analysis.

### Immune microenvironment landscape and drug sensitivity analysis

The estimation of stromal and immune cells was evaluated using the ESTIMATE algorithm. The stromal, immune, and ESTIMATE scores of ccRCC were estimated using the R package “estimate”. CIBERSORT algorithm was utilized to estimate the fraction of 22-type immune cells based on “CIBERSORT R script v1.03”. A single sample gene set enrichment analysis (ssGSEA) algorithm was performed to assess the proportion of 23-types of immune cells *via* the “GSVA” R package. The immune function score of each patient was estimated *via* the “GSVA” R package. Tumor Immune Dysfunction and Exclusion (TIDE) scores of each ccRCC sample were analyzed *via* the TIDE database (http://tide.dfci.harvard.edu/login/). Drug sensitivity (IC50) was a vital indicator for evaluating drug response to treatment. Based on the Genomics of Drug Sensitivity in Cancer (GDSC) database, the antineoplastic drugs response of each ccRCC sample in the low- and high-risk was predicted *via* R package “pRRophetic.” All statistical analyses were visualized *via* the “ggplot2” R package.

### Differential expression analysis and functional enrichment analysis

The R package “limma” was conducted to identify the differential expression genes (DEGs) in low- and high-risk group with the threshold set at |Fold Change| ≥ 2 and *P*-value < 0.05. Gene Ontology (GO) and Kyoto Encyclopedia of Genes and Genomes (KEGG) analysis were utilized to enrich the DEGs into the biological process and signaling pathways using the “clusterProfiler” R package (Yu et al., 2012). The activity of Hallmark terms of each ccRCC sample was conducted using R package “GSVA”.

### Statistical analysis

In this study, all statistical analysis were performed using the R software (version 4.1.0) and Perl scripts. Spearman-ranked correlation analysis was used to evaluate the association of the prognostic ARGs and immune cells, with *P*-value < 0.05 was considered significantly different. Differential functions were analyzed using the Wilcoxon rank-sum test between the two groups, and statistical significance was set at *P*-value < 0.05.

## Results

### Risk model construction based on the anoikis-related genes prognostic signature in clear cell renal cell carcinoma

**FIGURE 1:**
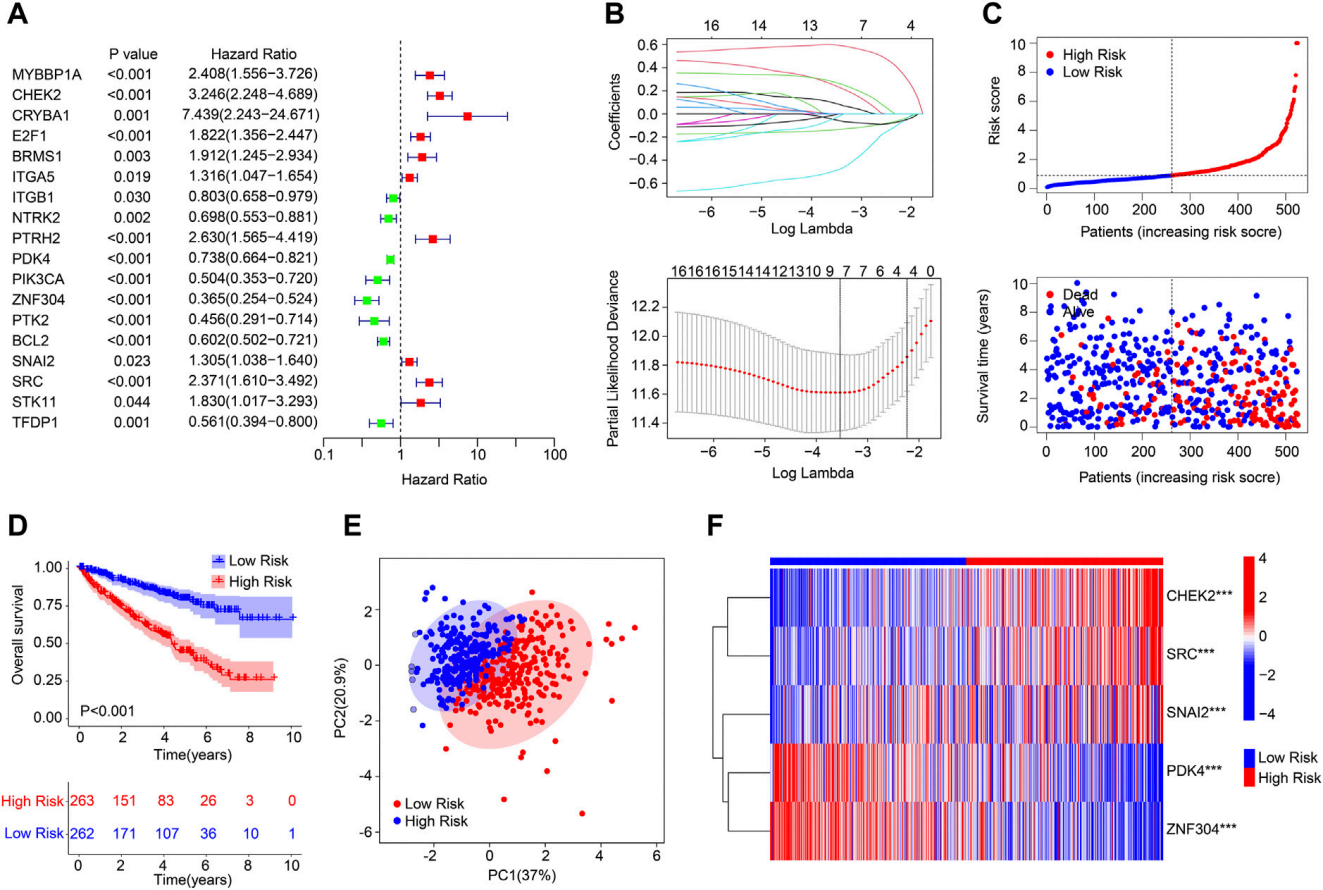
Risk model construction based on the ARGs prognostic signature in ccRCC. **(A)** Univariate Cox regression analysis of the ARGs. **(B)** LASSO regression analysis shows the minimum lambda and optimal coefficients of the prognostic ARGs. **(C)** Distribution of the ARGs prognostic signature and the scatter dot plots shows the correlation of the risk score and survival time. **(D)** The Kaplan-Meier survival curve analysis shows the OS rate of patients in the low- and high-risk group. **(E)** Principal component analysis shows a significant distribution of patients in the low- and high-risk group based on the ARGs prognostic signature. **(F)** Heatmap diagram displays the expression of the prognostic ARGs in the low- and high-risk group.

A novel risk model was developed to evaluate the prognostic value of ARGs in ccRCC. As shown in Figures 1A,B, according to the univariate Cox regression analysis, 9 ARGs associated with the OS rate were identified *via* the least absolute shrinkage and selection operator (LASSO) analysis. Based on the multivariate Cox regression analysis, 5 ARGs which could independently predict the prognosis for ccRCC were selected to establish the risk model. The ccRCC patients were ranked according to the median risk score and divided into the low- and high-risk group. The scatter dot plot suggested that the risk score was inversely correlated with the survival time for ccRCC (Figure 1C). Kaplan-Meier survival curve analysis illustrated that the OS rate of patients with the low-risk score was significantly higher compared to those of patients with high-risk score (Figure 1D). Principal component analysis (PCA) result illustrated a remarkable separation of patients in the low- and high-risk group based on the ARGs prognostic signature (Figure 1E). The expression of the 5 prognostic ARGs in the low- and the high-risk group were visualized in a heatmap diagram, and the results showed that the patients with high-risk score had higher expression of *CHEK2*, *SRC*, and *SNAI2*, whereas the expression of *PDK4* and *ZNF304* were higher in the low-risk group (Figure 1F).

### Validation of the anoikis-related genes prognostic signature in training cohort and test cohort

**FIGURE 2:**
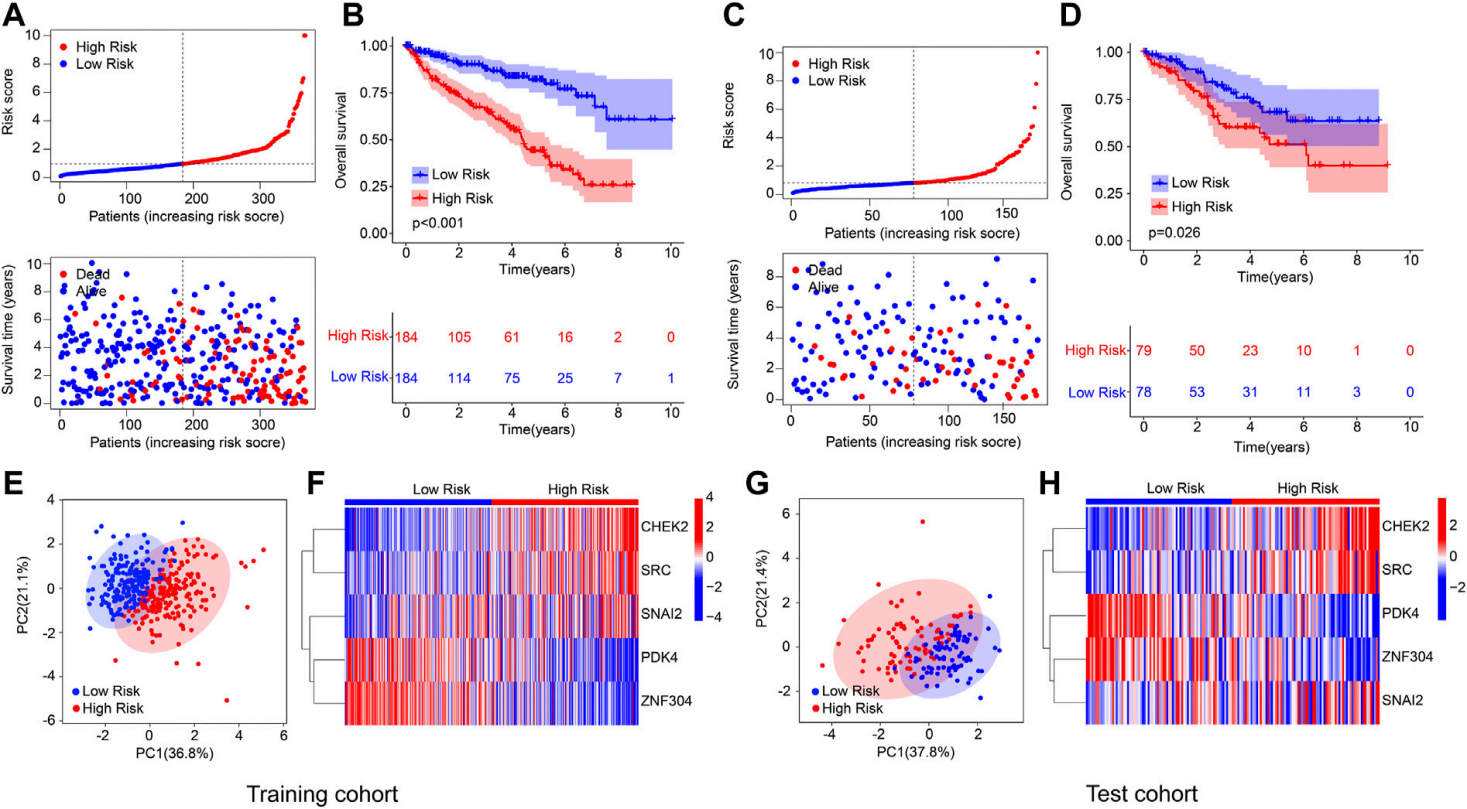
Risk model construction based on the 5 prognostic ARGs of ccRCC patients in the training cohort and test cohort. **(A)** Distribution of the ARGs prognostic signature and the correlation analysis between the survival time and prognostic signature in training cohort. **(B)** Kaplan-Meier survival cure analysis of patients with low- and high-risk score in training cohort. **(C)** Distribution of the ARGs prognostic signature and the correlation analysis between the survival time and prognostic signature in test cohort. **(D)** Kaplan-Meier survival cure analysis of patients with low- and high-risk score in test cohort. **(E)** PCA analysis of patients with ccRCC in training cohort based on the ARGs prognostic signature. **(F)** Heatmap diagram shows the expression of the 5 prognostic ARGs in training cohort. **(G)** PCA analysis of patients with ccRCC in test cohort based on the ARGs prognostic signature. **(H)** Heatmap diagram shows the expression of the 5 prognostic ARGs in test cohort.

An internal validation was conducted to evaluate the accuracy and independence of the ARGs prognostic signature in predicting the prognosis for patients with ccRCC. The patients with ccRCC were randomly classified into the training cohort and test cohort with a ratio was 7:3. According to the ARGs prognostic signature, the patients were ranked and classified into the low- and high-risk group in both cohorts. As shown in Figures 2A,C, the scatter dot plot illustrated that the risk score was inversely associated with survival time for patients in the training cohort and test cohort. Kaplan-Meier survival curve results indicated that the patients with low-risk score had higher OS rate compared to those with high-risk score in both cohorts (Figures 2B,D). The results of PCA suggested that the ARGs prognostic signature could clearly distinguish the patients ccRCC in the low- and high-risk group based on the ARGs prognostic signature in both cohorts (Figures 2E,G). The heatmap diagram suggested that the expression of *CHEK2*, *SRC*, and *SNAI2* were significantly higher in the high-risk group, but the expression of *PDK4* and *ZNF304* were lower in the high-risk group in both cohorts (Figures 2F,H). These results demonstrate that the risk model construction based on the ARGs prognostic signature could accurately evaluate the prognosis of patients with ccRCC.

### Kaplan-Meier survival analysis of anoikis-related genes prognostic signature in different clinicopathological characteristics

**FIGURE 3:**
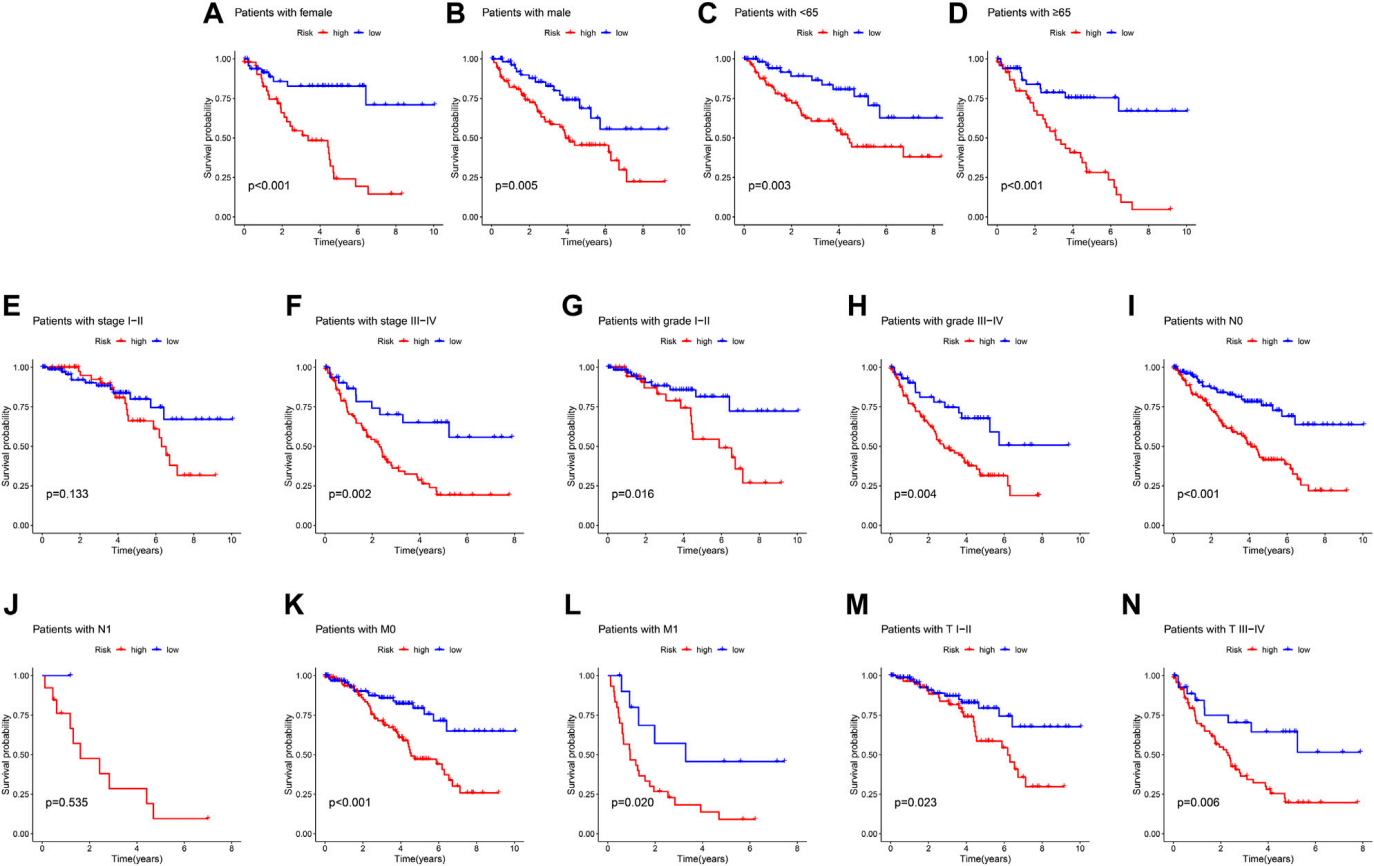
Correlation analysis of the ARGs prognostic signature and different clinicopathological characteristics. The OS rate of patients with ccRCC in the low- and high-risk group among the **(A)** Female; **(B)** Male; **(C)** Age <65; **(D)** Age ≥65; **(E)** Stage I-II; **(F)** Stage III-IV; **(G)** Grade I-II; **(H)** Grade III-IV; **(I)** N 0; **(J)** N 1; **(K)** M 0; **(L)** M 1; **(M)** T I-II; **(N)** T III-IV.

A classification subgroup analysis was performed to investigate the prognostic value of the ARGs prognostic signature in different clinicopathological characteristics. According to the ARGs prognostic signature, the patients with ccRCC were classified into the low- and high-risk group among the different clinicopathological characteristics. As shown in Figure 3, the Kaplan-Meier survival curve analysis suggested that the OS rate of patients with the low-risk score was significantly higher compared to those patients with high-risk group in gender (female vs. male), age (age <65 vs. age ≥65), stage III-IV, grade (grade I-II vs. grade III-IV), N 0, M (M 0 vs. M1), T (TI-II vs. T III-IV), whereas due to the sample size of patients in stage I-II and N1, the OS rate in stage I-II and N 1 was similar of the patients. These results demonstrate that the risk score based on the ARGs could accurately evaluate the prognosis of ccRCC patients relative to clinicopathological characteristics.

### Risk model based on the anoikis-related genes prognostic signature was an independent prognosis indicator

**FIGURE 4:**
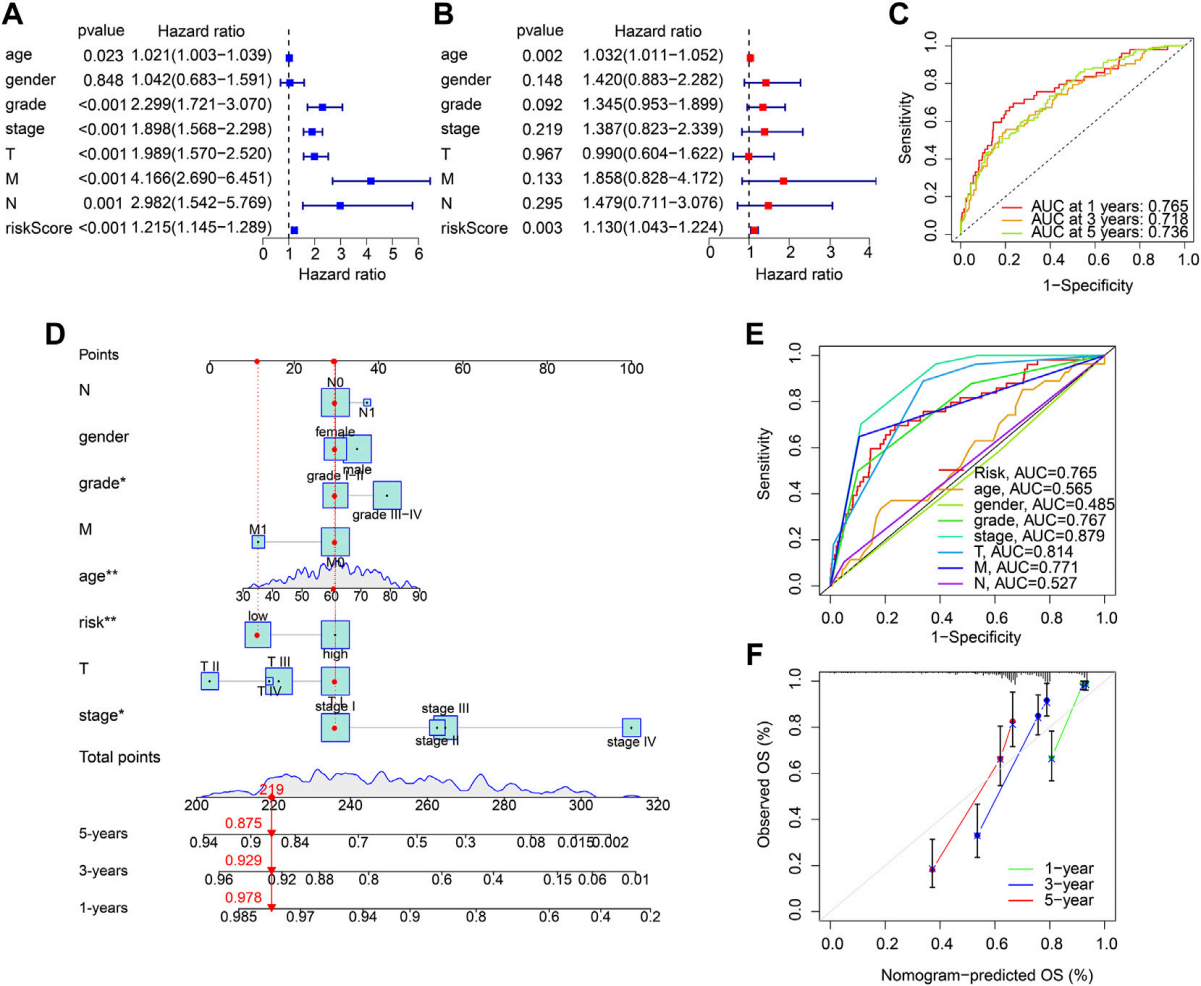
Independent prognosis analysis of the ARGs prognostic signature. **(A)** Univariate Cox regression analysis and **(B)** multivariate Cox regression analysis shows the correlation of the OS rate and risk score, and clinicopathological characteristics. **(C)** Time-dependent ROC curve shows the AUC at 1-, 3-, and 5-year **(D)** Nomogram construction based on the ARGs prognostic signature and clinicopathological characteristics. **(E)** ROC curve shows the accuracy of the risk score and clinicopathological characteristics. **(F)** Calibration curve shows the accuracy of the nomogram-predicted OS and actual OS.

Univariate and multivariate Cox regression analyses were utilized to evaluate the risk score based on the ARGs as an independent prognosis predictor for ccRCC. Univariate Cox regression analysis showed that age (hazard ratio (HR) = 1.021, *p* = 0.023), grade (HR = 2.299, *p* < 0.001), stage (HR = 1.898, *p* < 0.001), T (HR = 1.989, *p* < 0.001), M (HR = 4.166, *p* < 0.001), N (HR = 2.982, *p* = 0.001), and risk score (HR = 1.215, *p* < 0.001) were closely correlated with OS rate in ccRCC (Figure 4A). Multivariate Cox regression analysis result indicated that age (HR = 1.032, *p* = 0.002) and risk score (HR = 1.130, *p* = 0.003) were an independent prognosis indicator for ccRCC (Figure 4B). The time-dependent ROC curve showed that the AUC of 1-, 3-, and 5- years was 0.765, 0.718, and 0.736, respectively (Figure 4C). A novel nomogram model was established to accurately predict the 1-, 3-, and 5-year survival probability of ccRCC based on the ARGs signature and clinicopathological characteristics (Figure 4D). The ROC curve showed that the AUC of risk score was 0.765, suggesting a satisfactory stability of the ARGs prognostic signature (Figure 4E). The calibration curve indicated that the 1-, 3-, and 5-year’s OS rate predicted by nomogram was consisted with the actual OS rate (Figure 4F). These results demonstrate that the risk score based on the ARGs is an independent prognosis predictor and could accurately evaluate the survival probability of ccRCC patients relative to clinicopathological characteristics.

### Functional enrichment analysis of the differential expression genes

**FIGURE 5:**
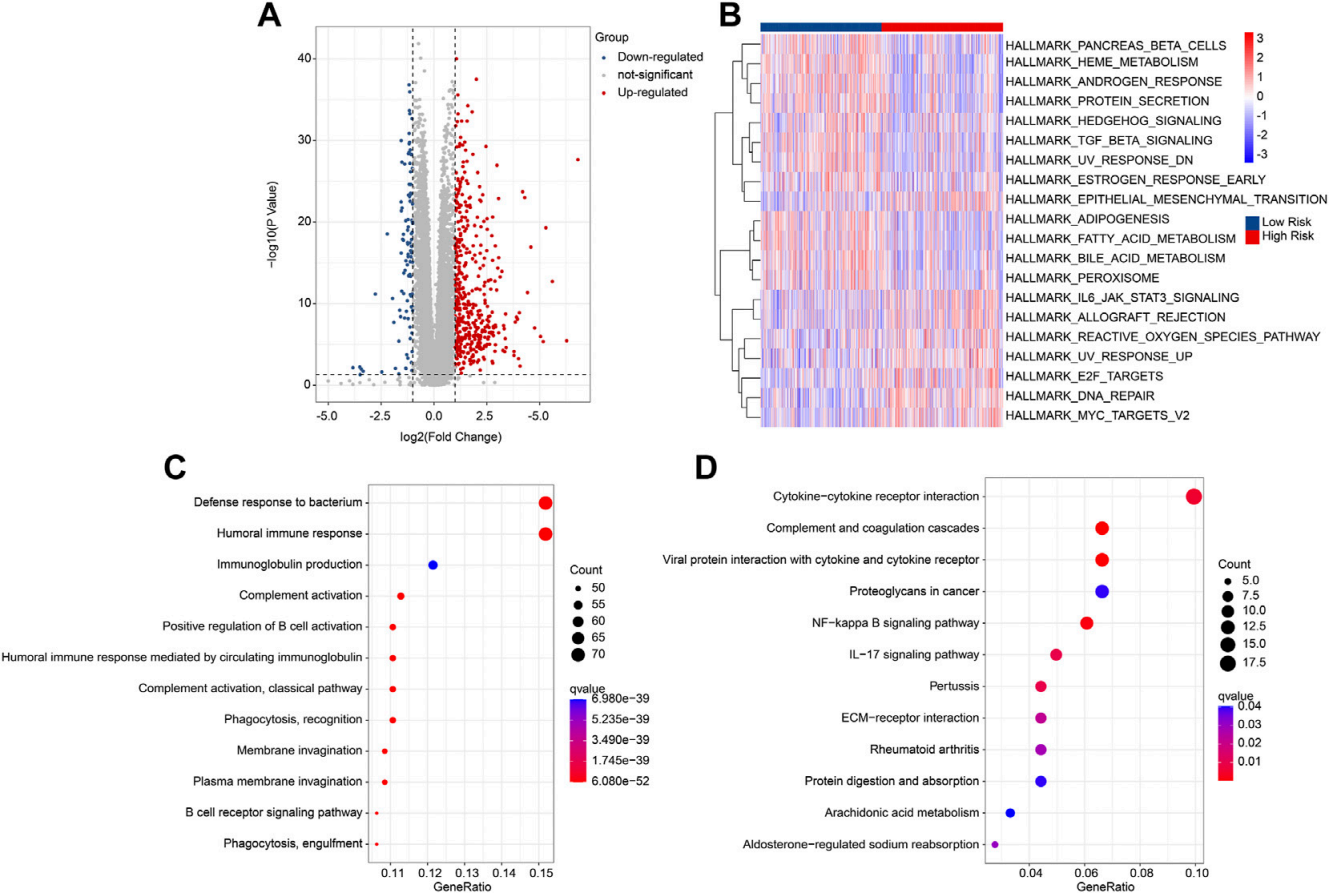
Functional enrichment analysis of DEGs in the low- and high-risk group. **(A)** Volcano diagram shows the DEGs with the threshold set at |FC| ≥ 2 and *P*-value < 0.05. **(B)** GSVA analysis of hallmark signaling pathway for each ccRCC patient in the low- and high-risk group. **(C)** GO enrichment analysis shows the biological process of DEGs. **(D)** KEGG enrichment analysis shows the enrichment signaling pathways of DEGs.

Multiple enrichment methods were utilized to investigate the potential molecular mechanism of DEGs in the low- and high-risk group. The DEGs in the low- and high-risk groups were illustrated in a volcano diagram, and the result showed that most of DEGs were upregulated in the high-risk group (Figure 5A). GSVA analysis results illustrated the hallmark signaling pathways of the DEGs for each patient in the low- and high-risk group (Figure 5B). GO enrichment analysis revealed that the DEGs were enriched in immune-related biological processes, such as defense response to bacterium, humoral immune response, and immunoglobulin production (Figure 5C). KEGG analysis result suggested that cytokine-cytokine receptor interaction was significantly enriched of the DEGs (Figure 5D). These findings demonstrate that immune-related signaling pathways may mediate the role of the ARGs in tumourigenesis of ccRCC.

### Consensus clustering and immune microenvironment landscape analysis

**FIGURE 6:**
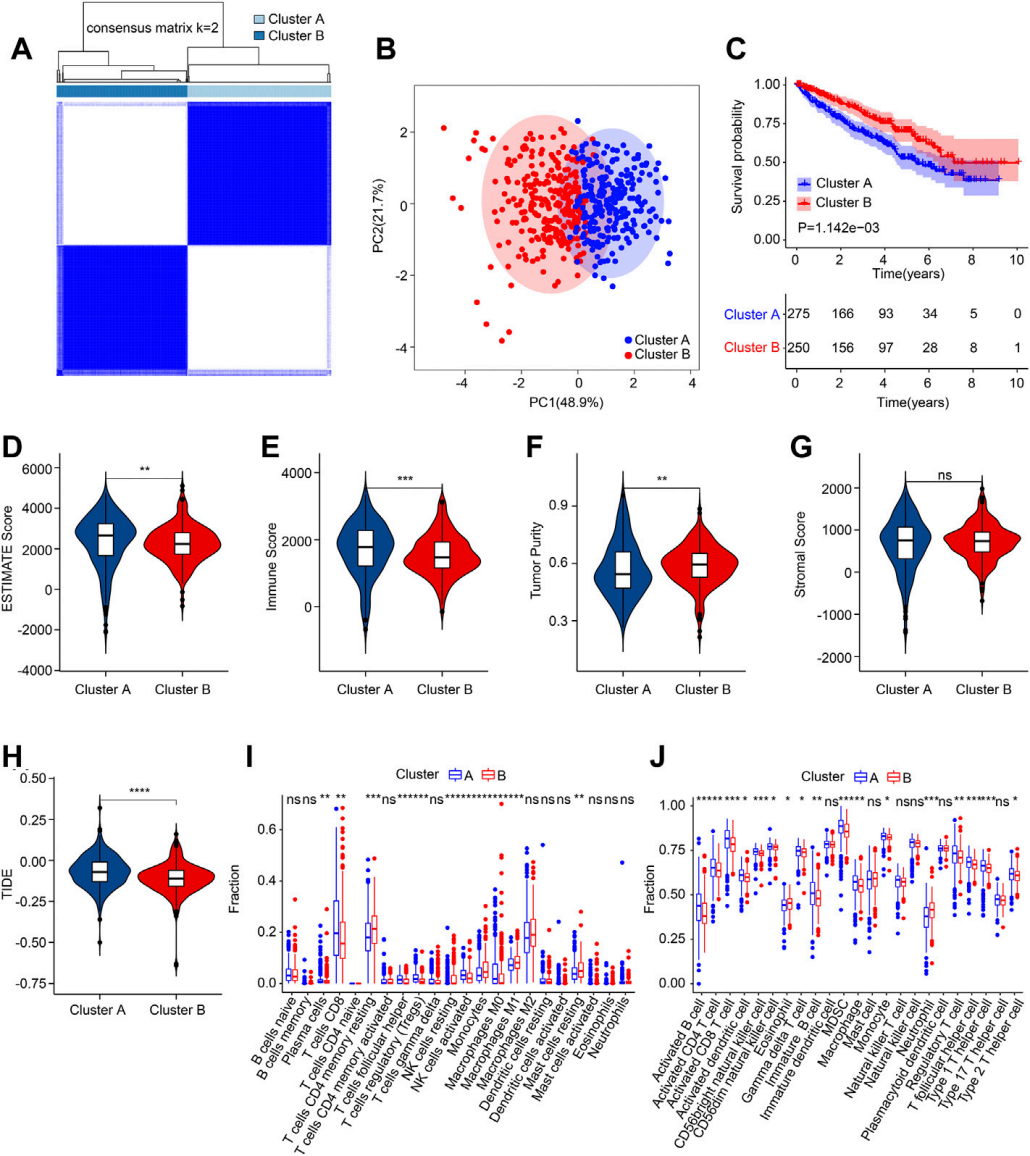
Consensus clustering of ccRCC patients and immune microenvironment landscape analysis. **(A)** Consensus clustering heatmap shows the optimal classification of ccRCC samples with K = 2. **(B)** PCA analysis shows a significant distribution pattern of patients in Cluster A and Cluster B. **(C)** The Kaplan-Meier survival curve shows the OS rate of patients in Cluster A and Cluster B. **(D)** ESTIMATE score. **(E)** Immune score. **(F)** Tumor purity. **(G)** Stromal score. **(H)** TIDE score. **(I)** The fraction of 22-type immune cells in low- and high-risk group. **(J)** The proportion of 23-type immune cells in low- and high-risk group.

Consensus clustering analysis was employed to cluster the ccRCC patients into different subgroups based on the 5 prognostic ARGs. The heatmap showed an optimal classification of the ccRCC patients with the K = 2, with 275 samples in Cluster A and 250 samples in Cluster B (Figure 6A). The PCA score plot illustrated a remarkable separation between Cluster A and Cluster B based on the 5 prognostic ARGs (Figure 6B). The Kaplan-Meier survival curve indicated that the patients in Cluster A had a lower OS rate than those patients in Cluster B (Figure 6C). The ESTIMATE algorithm was utilized to investigate the immune microenvironment landscape in Cluster A and Cluster B, and the results showed that the patients in the Cluster had higher ESTIMATE, immune scores, but lower tumor purity (Figures 6D–G). TIDE result revealed that the patients in Cluster B had lower TIDE score, suggesting a better potential immunotherapy response for ccRCC patients in the Cluster B (Figure 6H). Moreover, ssGSEA and CIBERSORT algorithms were performed to evaluate the immune infiltration landscape of patients with ccRCC in Cluster A and Cluster B. As shown in Figure 6I, the CIBERSORT algorithm showed that the proportion of T cells CD8, Plasma cells, T cells follicular helper, T cells regulatory (Tregs), NK cells activated, and macrophages M0 were higher in patients in Cluster A, whereas the fraction of T cells CD4 memory resting, NK cells resting, monocytes, macrophages M1, and mast cells resting were higher of patients in Cluster B. The result of ssGSEA suggested that the proportion of most immune cells were significantly higher in patients in Cluster a, but the proportion of eosinophil and neutrophil were lower in patients in Cluster A (Figure 6J). Collectively, these results illustrate that the ARGs are associated with prognosis and could indicate the immune response and immune infiltration landscape in ccRCC.

### Correlation analysis of the anoikis-related genes prognostic signature and immune infiltration landscape

**FIGURE 7:**
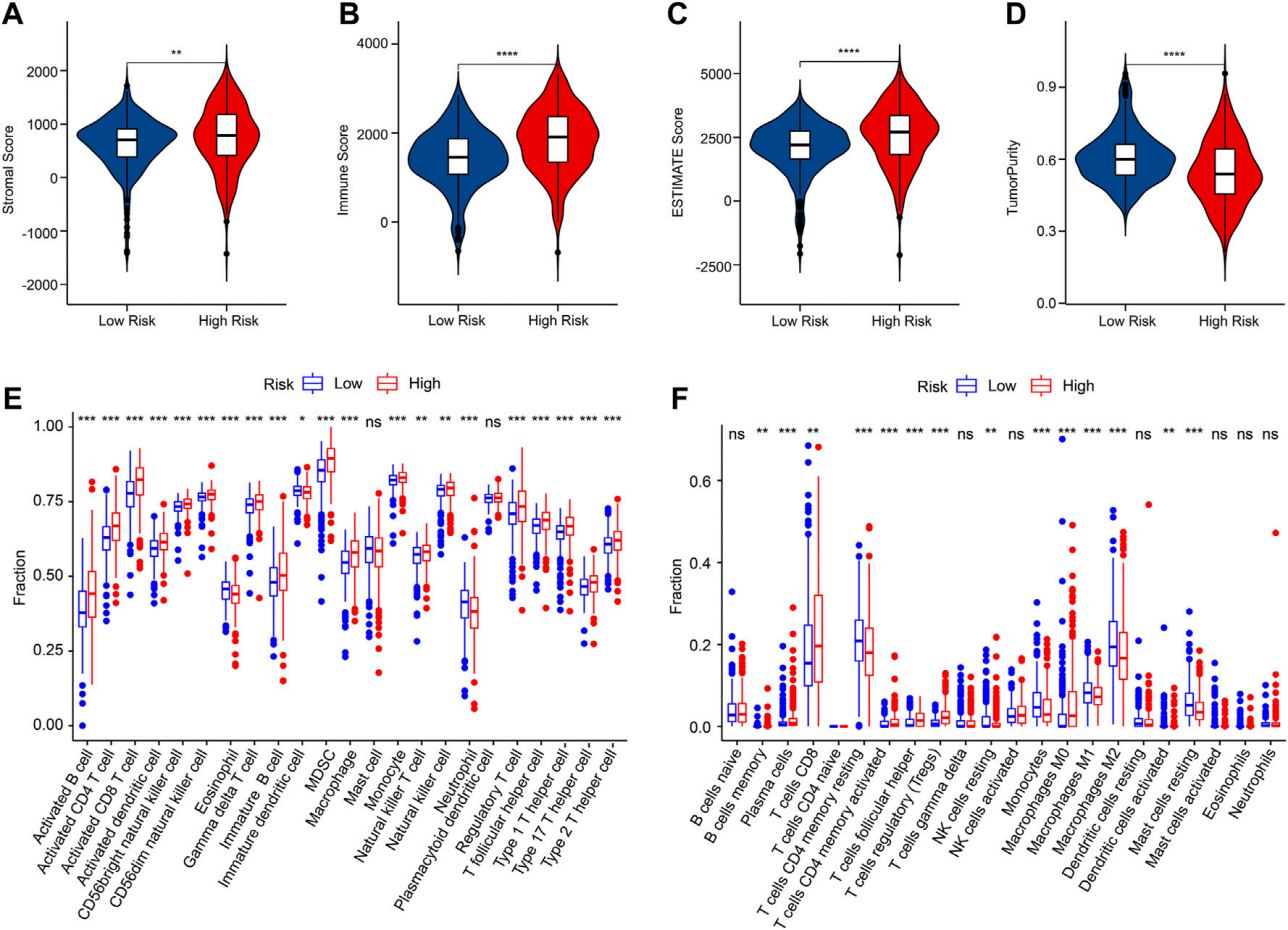
Immune infiltration landscape of patients in the low- and high-risk group. **(A–D)** Stromal, immune, ESTIMATE scores and tumor purity. **(E)** The fraction of 23-type immune cells of patients in the low- and high-risk group. **(F)** The proportion of 22-type immune cells of patients in the low- and high-risk group.

Multiple immune assessment algorithms were employed to estimate the immune infiltration landscape of patients in the low- and high-risk group. The ESTIMATE results showed higher stromal, immune, and ESTIMATE scores, and lower tumor purity of patients in the low-risk group (Figures 7A–D). ssGSEA algorithm result suggested that the fraction of most immune cells was significantly higher in the high-risk group, whereas the fraction of eosinophil, immature dendritic cell, and neutrophil were higher in the low-risk group (Figure 7E). The CIBERSORT result revealed that the patients with low-risk score had higher proportion of T cells CD4 memory resting, NK cells resting, monocytes, macrophages M1, macrophages M2, dendritic cells activated, and mast cells resting, but lower proportion of B cells memory, plasma cells, T cells CD8, T cells CD4 memory activated, T cells follicular helper, T cells regulatory (Tregs), and macrophages M0 (Figure 7F).

**FIGURE 8:**
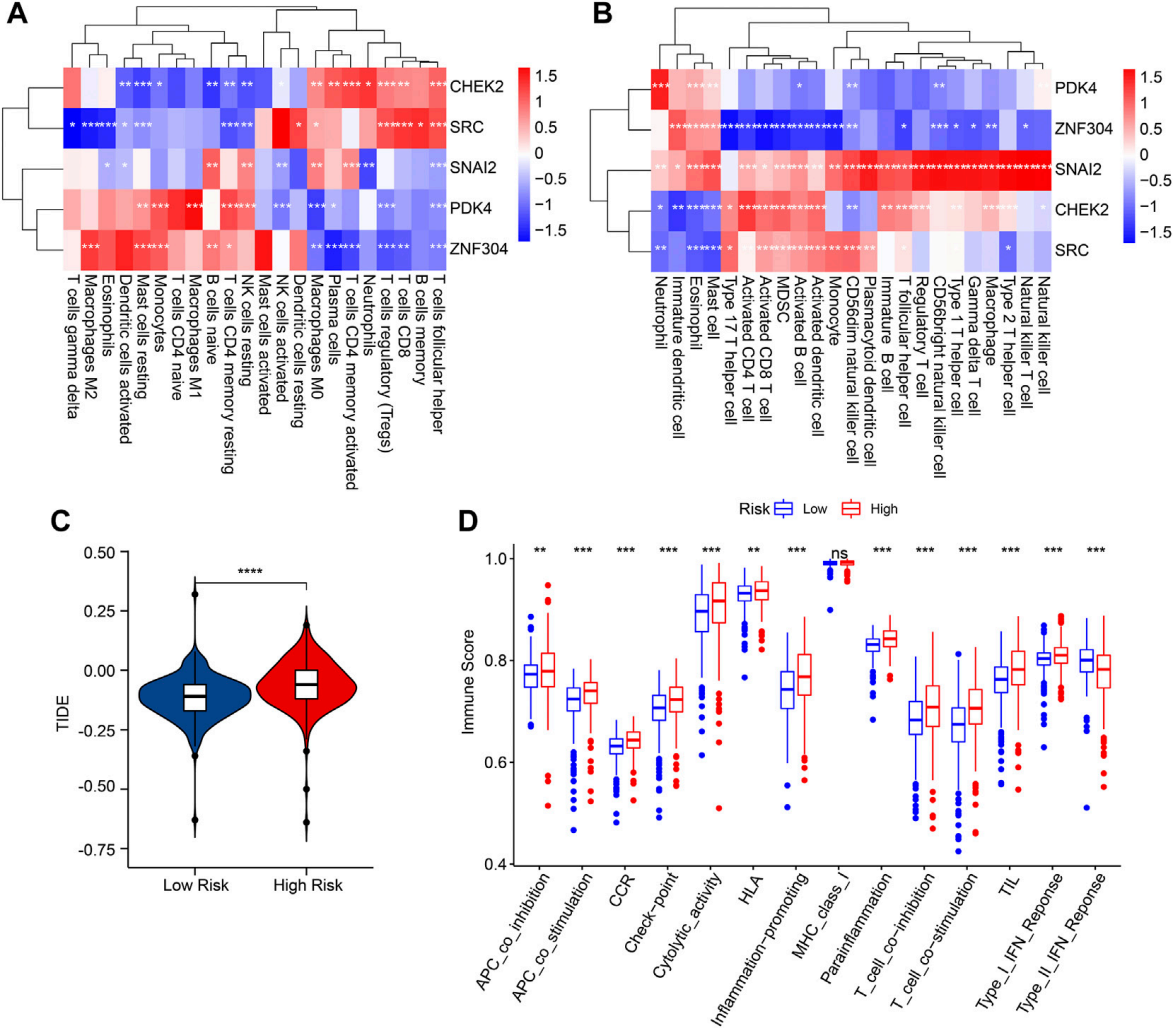
Correlation analysis of the prognostic ARGs and immune infiltration landscape. **(A,B)** The heatmap shows the correlation of the prognostic ARGs and immune cells. **(C)** TIDE score. **(D)** Immune function score.

Correlation analysis was conducted to investigate the association between prognostic ARGs and immune infiltration landscape. The correlation analysis result showed a remarkable association between prognostic ARGs and 22-type immune cells as calculated by CIBERSORT, such as *CHEK2* and *SRC* were positively correlated with T cells follicular helper, T cells CD8, and macrophages M0 (Figure 8A). Moreover, *ZNF304* was negatively correlated with most of the 23-type immune cells, but positively correlated with neutrophil, eosinophil, and mast cell; *SNAI2* was positively associated with the 23-type immune cells; *CHEK2* and *SRC* were positively correlated with most of the 23-type immune cells (Figure 8B). Considering the remarkable difference in immune infiltration landscape for ccRCC patients, the response to immunotherapy was further evaluated of patients in the low- and high-risk group. TIDE result revealed that the patients with low-risk score had lower TIDE score, suggesting a better response to immunotherapy of patients in the low-risk group (Figure 8C). The immune function score result showed that the patients with high-risk score had higher immune function score, such as cytolytic activity, check point, and HLA (Figure 8D). Taken together, these results demonstrate that the risk model based on the ARGs prognostic signature is correlated with the immune infiltration landscape and immunotherapy response of patients with ccRCC.

### Drug sensitivity analysis

**FIGURE 9:**
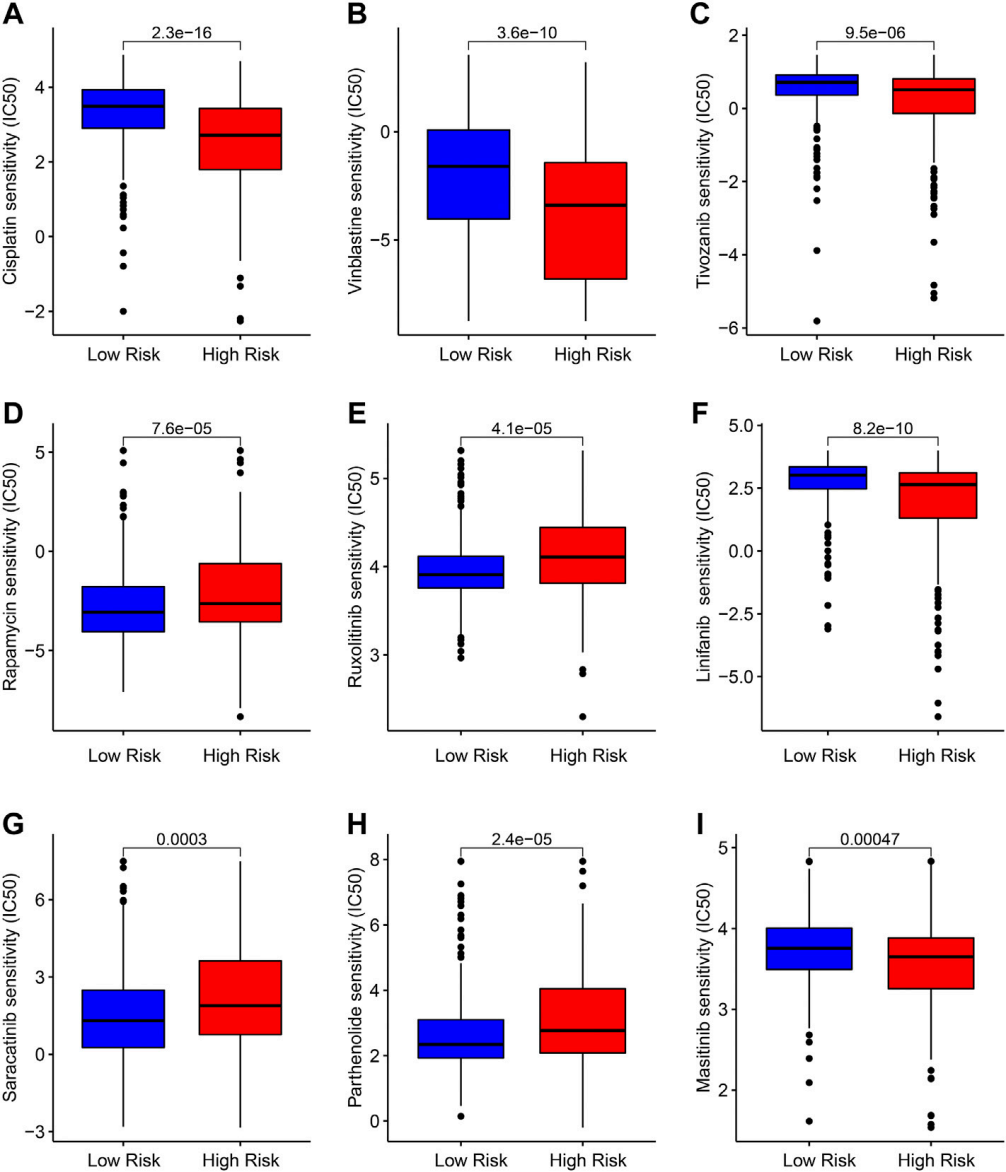
Drug sensitivity analysis of patients in the low- and high-risk group. Distribution of IC50 values in the low- and high-risk group among **(A)** Cisplatin, **(B)** Vinblastine, **(C)** Tivozanib, **(D)** Rapamycin, **(E)** Ruxolitinib, **(F)** Linifanib, **(G)** Saracatinib, **(H)** Parthenolide, and **(I)** Masitinib.

Targeted therapy is a vital strategy in the clinical management of ccRCC. In the subsequent analysis, several potential antineoplastic drugs were identified which may benefit the treatment of ccRCC. As shown in Figure 9, the drug sensitivity analysis results suggested that the IC50 of Cisplatin, Vinblastine, Tivozanib, Linifanib, and Masitinib were significantly higher in the low-risk group, whereas the IC50 of Rapamycin, Ruxolitinib, Saracatinib, and Parthenolide were higher in the high-risk group. These above results demonstrate a promising response to the antineoplastic drug of patients with ccRCC in different risk subgroups, providing a novel insight into the precisely targeted therapy for ccRCC patients.

## Discussion

Since the prognosis for metastatic or advanced ccRCC patients remains unsatisfactory, early diagnosis and risk stratification for improving the survival time of patients with ccRCC is essential. Here, 5 ARGs were identified as being associated with OS rate for ccRCC, and a novel risk model was established to successfully evaluate the prognosis of ccRCC. Involved immune infiltration landscape and drug sensitivity analysis were further evaluated.

As a tumor suppressor protein that plays a role in the p53 signaling pathway, *CHEK2* has been reported to be associated with carcinogenesis in several tumor types, including RCC (Boonen et al., 2022). Several studies have demonstrated an association between *CHEK2* germline mutations and RCC. In a NGS sequencing study of 254 patients with advanced RCC, 41 carriers of pathogenic germline mutations in kidney cancer or other cancer-related genes were identified (Carlo et al., 2018). Of these, the *CHEK2* germline mutation found in 9 patients (3.4%) exceeded the most common change in RCC-related mutations. Similar results were obtained in another study, which identified 7 out of 229 (3.1%) mutation carriers with germline *CHEK2* variants in patients with metastatic ccRCC (Ged et al., 2020). In patients with early-onset RCC, *CHEK2* germline mutation was also the most common change found before the age of 60 years (19/844) (Hartman et al., 2020). Although there is now increasing evidence that *CHEK2* germline mutations are associated with an increased risk of RCC, larger case-control studies in patients with RCC are needed to confirm and refine the magnitude of the associated risk (Stolarova et al., 2020).

*SRC* encodes a tyrosine-protein kinase and has shown its impact on the regulation of embryonic development and cell growth. In RCC, *SRC* leads to distal lung metastasis through glycolytic reprogramming (Zhang et al., 2021). Furthermore, *SRC* contributes to the emergence of malignant phenotypes in renal cancer cells, particularly due to the resistance of BCL-XL to apoptosis and angiogenesis stimulated by SRC-STAT3-VEGF signaling (Chatterjee et al., 2022). These results suggest that *SRC* contributes to the emergence of malignant phenotypes in renal cancer cells, which are in line with our data that *SRC* is highly-expressed in high-risk group. Concerning the protein encoded by this gene is a tyrosine-protein kinase, further research on *SRC* has the potential for clinical application.

*SNAI2* encodes a member of the Snail family of C2H2-type zinc finger transcription factors, which is involved in epithelial-mesenchymal transitions (EMT) and has antiapoptotic activity (Shang et al., 2022). Our data showed *SNAI2* high expression was correlated with worse outcomes in RCC patients. In the carcinogenesis process, *SNAI2* has been reported to take active part in metastasis, progression, differentiation, and drug sensitivity in multiple cancer types (Jin et al., 2022; Mazzu et al., 2022; Sorin et al., 2022). In ccRCC, by facilitates the EMT, *SNAI2* promotes cancer cell migration and invasion (Jiang et al., 2019). Since EMT has been shown to be an important factor in tumor progression, its facilitator *SNAI2* may have an even more important role in RCC carcinogenesis (Fiori et al., 2019).

*PDK* is the enzyme responsible for phosphorylating pyruvate dehydrogenase and the metabolic switch from mitochondrial respiration to cytoplasmic glycolysis (Heinemann-Yerushalmi et al., 2021; Querfurth et al., 2022). *PDK4* is decreased in a variety of cancers, such as gastric cancer, prostate cancer, breast cancer, lung cancer and liver cancer, and may be associated with the inhibition of cell proliferation and induction of apoptosis (Liu et al., 2021). The function of *PDK4* has not been previously reported in RCC. Our data show similar results in RCC. More recently, it has been suggested that this switch plays a key role in increasing drug resistance. By reprogramming drug metabolism, *PDK4* has been reported to modulate chemoresistance, including 5-fu and cisplatin (Woolbright et al., 2018; Wang J. et al., 2019; Yu et al., 2021). The differences in drug resistance between subgroups we demonstrated may be related to metabolic differences due to differences in *PDK4* expression levels.

*ZNF304* plays a key role in the regulation of cell survival, proliferation, apoptosis, and differentiation during development by transcriptional silencing of genes. As one of the key anoikis players, *ZNF304*-integrin axis has been shown to fight against anoikis during tumor development and promote a variety of proto-cancer pathways important for cell survival, migration, and invasion in ovarian cancer (Aslan et al., 2015). However, in contrast, our data showed the expression of *ZNF304* was relatively low in high-risk ccRCC patients. Lower levels of *ZNF304* were associated with poorer survival. *In vitro* experiments also showed that down-regulation of *ZNF304* affected mir-183-5p/FOXO4 axis and further inhibited cell growth in ccRCC, while overexpression of *ZNF304* inhibited growth (Ren et al., 2021). Given the contradictory roles of its target, mir-183-5p, in different tumor types, this may depend on the biological function differences of the targets in different cancer species.

RCC is considered to be an immunogenic tumor, and a large number of immune cells, such as tumor-infiltrating lymphocytes, can be detected in the tumor tissue (Nakayama et al., 2018). Therefore, the use of immunotherapy to produce an effective immune response to the tumor to delay the development of cancer is considered to be effective in RCC (Sendur, 2022). According to our immune infiltration analysis results, high-risk RCC had an immune microenvironment consisting of higher levels of CD8^+^ T cells, CD4^+^ T cells, and lower M2 macrophages. It is also suggested that immunotherapy is more beneficial in high-risk RCC patients. In addition, in our results, immunosuppressive cells (MDSC and Treg) were significantly elevated in the high-risk group. Multiple therapeutic approaches have provided evidence of immune priming in RCC by reducing Treg levels and have been used in the clinical. Antiangiogenic agents have been shown to delay tumor progression not only by impounding angiogenesis in the tumor microenvironment, but also by suppressing the immune response of immunosuppressive cytokines and cells, such as Treg cells (Tartour et al., 2011; Doleschel et al., 2021). TKI also causes the immune initiation of Treg decline through the regulation of VEGF (Tallima et al., 2021). Therefore, our results support that patients in the high-risk group may benefit more from immunotherapy.

Extracellular matrix (ECM) is essential for various biological functions during tumor progression, including the induction of anoikis resistance and cell adhesion- mediated drug resistance (Wang et al., 2022). Our data show significant differences in susceptibility to chemotherapeutic agents in different risk stratifications after risk modeling with anoikis-associated genes. Additionally, in this study, TIDE analysis was used to test the interaction between candidate genes and cytotoxic T cell function and the extent to which it affects the risk of death. The high-risk group had higher TIDE levels. Higher tumor TIDE prediction scores were associated not only with poor immune checkpoint suppression therapy but also with poor patient survival under anti-PD1 and anti-CTLA4 therapy (Chen et al., 2021). In patients with advanced RCC, the combination of ipilimumab (anti-CTLA-4 antibody) and nivolumab (anti-PD-1 antibody) was compared with the previous sunitinib (VEGF-TKI), resulting in a significant improvement in treatment. However, more than half of the patients could not achieve the long-term response to PD-1-related treatment (Motzer et al., 2018). Therefore, it is important to further screen suitable RCC patients for PD-1 treatment. In this study we have provided preliminary evidence for anoikis future *in vivo* and *in vitro* experiments will be necessary to further verify the efficacy of anoikis against immune checkpoint inhibitors.

In the present study, we established a novel model based on prognostic ARGs and preliminarily evaluated the efficacy of risk model in predicting the prognosis of ccRCC patients. We also preliminarily describe the guiding significance of the model for chemoresistance and immune-related therapy. In addition, immune infiltration landscape analysis and functional enrichment analysis were evaluated, which preliminarily demonstrated the association between the risk model and the immunosuppressive microenvironment. However, there are still some shortcomings in this study. The results based on bioinformatics in this paper are not verified by *in vitro* experiments. In the next step, we will experimentally verify the significance of anoikis in RCC. In conclusion, our findings provide novel insights and perspectives into a new potential therapeutic strategy and antitumor targets for ccRCC.

## Data Availability

The datasets presented in this study can be found in online repositories. The names of the repository/repositories and accession number(s) can be found in the article/Supplementary Material.
